# Tidal volumes during delivery room stabilization of (near) term infants

**DOI:** 10.1186/s12887-022-03600-y

**Published:** 2022-09-13

**Authors:** Janine Thomann, Christoph M. Rüegger, Vincent D. Gaertner, Eoin O’Currain, Omar F. Kamlin, Peter G. Davis, Laila Springer

**Affiliations:** 1 Department of Neonatology, Newborn Research, University Hospital and University of Zurich, Frauenklinikstrasse 10, 8091 Zurich, Switzerland; 2 School of Medicine, University College Dublin and National Maternity Hospital Dublin, Dublin, Ireland; 3 Newborn Research Centre and Neonatal Services, The Royal Women’s Hospital, Melbourne, Australia; 4 The University of Melbourne, Melbourne, Australia; 5 Murdoch Children’s Research Institute, Melbourne, Australia; 6 Department of Neonatology, University Clinic Tübingen, Tübingen, Germany

**Keywords:** Term and late preterm infants, Delivery room stabilization, Positive pressure ventilation, Tidal volume

## Abstract

**Background:**

We sought to assess tidal volumes in (near) term infants during delivery room stabilization.

**Methods:**

Secondary analysis of a prospective study comparing two facemasks used for positive pressure ventilation (PPV) in newborn infants ≥ 34 weeks gestation. PPV was provided with a T-piece device with a PIP of 30 cmH_2_O and positive end-expiratory airway pressure of 5 cmH_2_O. Expired tidal volumes (V_t_) were measured with a respiratory function monitor. Target range for V_t_ was defined to be 4 – 8 ml/kg.

**Results:**

Twenty-three infants with a median (IQR) gestational age of 38.1 (36.4 – 39.0) weeks received 1828 inflations with a median V_t_ of 4.6 (3.3 – 6.2) ml/kg. Median V_t_ was in the target range in 12 infants (52%), lower in 9 (39%) and higher in 2 (9%). Thirty-six (25—27) % of the inflations were in the target rage over the duration of PPV while 42 (25 – 65) % and 10 (3 – 33) % were above and below target range.

**Conclusions:**

Variability of expiratory tidal volume delivered to term and late preterm infants was wide. Reliance on standard pressures and clinical signs may be insufficient to provide safe and effective ventilation in the delivery room.

**Trial registration:**

This is a secondary analysis of a prospectively registered randomized controlled trial (ACTRN12616000768493).

## Background

Although most newborn infants initiate respiration spontaneously or after tactile stimulation within the first minutes after birth, approximately 5% require additional support [1]. Current guidelines recommend mask ventilation as the next step to achieve aeration of the lung [1, 2]. While volume-targeted ventilation has become standard of care in the neonatal intensive care unit due to its lung-protective effects [3], tidal volumes (V_t_) are not routinely measured during non-invasive positive pressure ventilation (PPV) in the delivery room. Studies in preterm infants suggest an association between excessive V_t_ and intraventricular hemorrhage [4]. Others suggest that low V_t_ are associated with a higher intubation rate [5]. A clinical trial comparing the use of a RFM during PPV of preterm infants in the delivery room as a guidance for V_t_ delivery with no RFM showed that only 30% of inflations were within the target range [6]. In term and late preterm infants, less is known about the variability of measured V_t_ and changes of V_t_ over time. A recent single center study reported a median tidal volume at the lower end of the recommended range and substantial variation in tidal volumes measured in term infants [7].

In spontaneously breathing newborn term infants, expired V_t_ are known to be highly variable, ranging from 0.5 to more than 20 ml/kg [8, 9]. This may be due to changing breathing patterns during the transitional period [10]. During the provision of PPV, expired Vt may also vary chiefly due to interaction between spontaneous breaths and mask inflations.

Current European Resuscitation (ERC) guidelines recommend providing initial peak inflation pressures (PIP) of 30 cm H_2_O for term infants [1]. These recommendations are based on limited evidence [11]. The primary aim of this study was to assess V_t_ measured in term and late preterm infants during delivery room stabilization and changes in applied V_t_ over the duration of PPV.

## Methods

### Population and intervention

This is a secondary analysis of a previously published randomized controlled trial conducted at the Royal Women’s Hospital in Melbourne, Australia (ACTRN12616000768493) [12]. The original study and secondary analyses were approved by the local ethics committee. All parents provided written informed consent. The original trial compared the effect of two facemasks on mask leak in newborn infants ≥ 34 weeks gestation receiving PPV immediately after birth. PPV was started according to local neonatal resuscitation guidelines if the infant was gasping, not breathing or had a heart rate < 100 min [13]. The original study showed that the suction mask may have negative effects on mask ventilation [12, 14]. Therefore, the current secondary analysis only included infants resuscitated with the conventional mask (Laerdal Silicone mask, Laerdal, Stavanger, Norway).

### Measurements and data collection

A Neopuff Infant Resuscitator (Fisher & Paykel Healthcare, Auckland, New Zealand) was used to provide PPV with initial peak inflation pressure (PIP) of 30 cm H_2_O and positive end-expiratory airway pressure (PEEP) of 5 cm H_2_O [1]. Changes to pressure settings were at the discretion of the treating clinician. Respiratory function parameters were recorded continuously during PPV using the NewLifeBox (Advanced Life Diagnostics UG, Weener, Germany). Breath-by-breath analysis was performed by manually placing inspiratory and expiratory markers using Pulmochart (Advanced Life Diagnostics UG). As previously published [6], all PPV inflations, including those coinciding with spontaneous breaths, were included. The clinical team was blinded to the RFM data. All resuscitations were video recorded.

### Statistical analysis

Data from all available breaths were analysed. Consistent with previous studies, we defined the optimal range for expiratory V_t_ to be 4 – 8 ml/kg [15]. Leak was calculated as the difference between inspiratory V_t_ and expiratory V_t_, and expressed as a percentage of inspiratory V_t _[16]. Obstruction was defined as V_t_ < 2 ml/kg with leak < 30% [16]. Variability of V_t_ was assessed by calculating the coefficient of variation (standard deviation divided by mean). The higher the coefficient of variation, the greater the level of dispersion around the mean. Skewed data are presented as median and interquartile range (IQR). Friedman test was used to compare V_t_ between different time points throughout episodes of PPV and Mann–Whitney U-test was used to compare parameters between groups. *P*-values < 0.05 were considered statistically significant. Analyses were performed using SPSS (IBM SPSS Statistics for Windows, Version 26.0. Armonk, NY: IBM Corp).

## Results

### Population

Overall, 23 infants with a median (IQR) gestational age of 38.1 (36.4 to 39.0) weeks received 1828 PPV inflations (see Table 1). A median of 110 inflations (67 to 181) were recorded per infant.

**Table 1:** Demographics and respiratory outcomes for *n* = 23 infants. Numbers are presented as median (IQR) or numbers (%)

**Demographics**	
Gestational age (weeks)	38.1 (36.4 – 39.0)
Birth weight (g)	3210 (2810 – 3330)
Male (%)	13 (57%)
Cesarean delivery (%)	21 (91%)
Maternal general anesthesia (%)	7 (30%)
Umbilical cord pH	7.20 (7.11 – 7.24)
Apgar 1 min	3 (2 – 4)
Apgar 5 min	8 (6 – 9)
Apgar 10 min	9 (8 – 9)
Meconium stained amniotic fluid	5 (22%)
Admission to NICU	10 (43%)
Intubation	2 (9%)
**Respiratory ouctomes**	
V_t_ (ml/kg)	4.6 (3.3 – 6.2)
PIP (mbar)	30.5 (29.9 – 31.8)
PEEP (mbar)	5.2 (4.2 – 5.8)
Respiratory rate (bpm)	51 (44 – 56)
Ti (s)	0.58 (0.53 – 0.66)

### Tidal volume

**Fig. 1:**
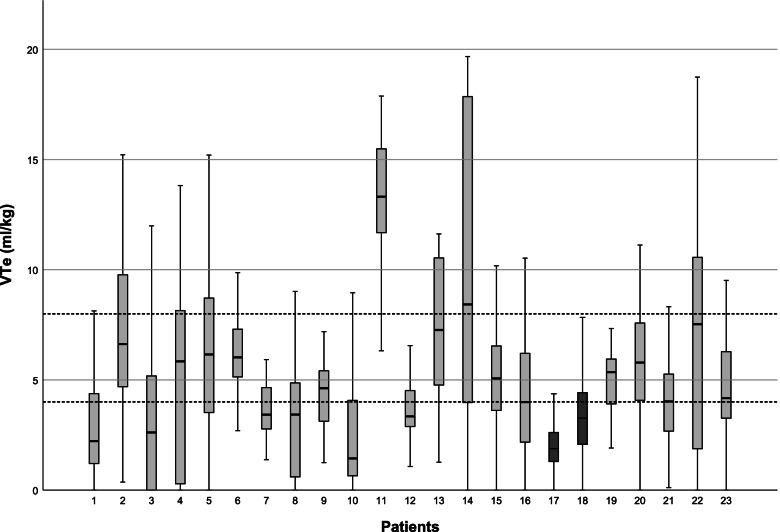
Shows V_t_ in ml/kg of each infant. Reference lines indicate a target range of 4 – 8 ml/kg. The two patients in dark grey (17 and 18) were the two infants who were intubated

**Fig. 2:**
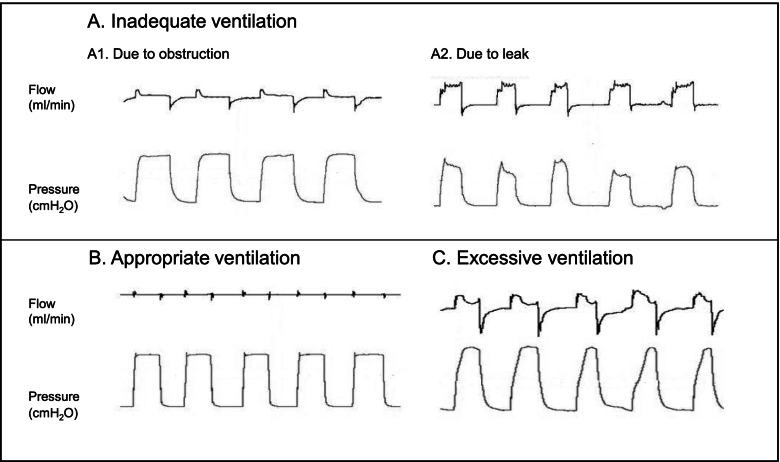
Shows flow and pressure wave forms as an example for inadequate ventilation (**A**), appropriate ventilation (**B**) and excessive ventilation (**C**). A1 shows inadequate ventilation due to obstruction: although pressure is applied, there is almost no airflow. A2 shows inadequate ventilation due to mask leak: there is very little airflow coming back during expiration. B is an example of appropriate ventilation: there is no leak and VTe is in the target range. C shows excessive ventilation

**Fig. 3:**
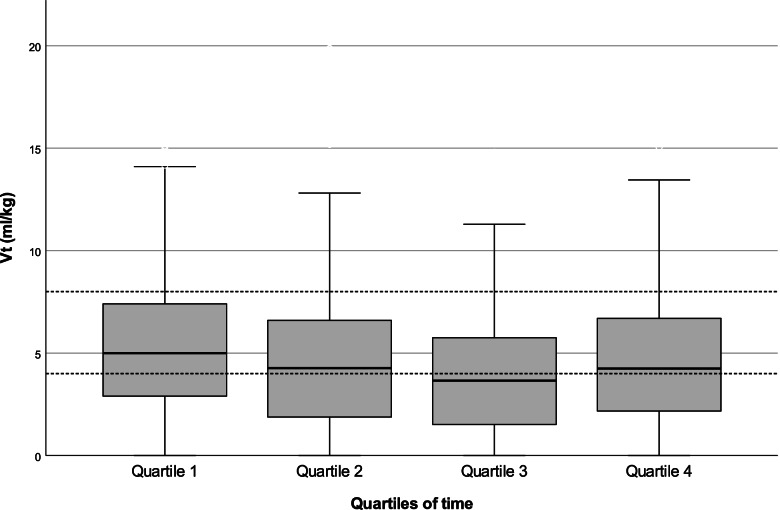
Shows development of Vt over time. Quartile 1 summarizes the first 25% of time of PPV, quartile 2 25–50% of time, quartile 3 50–75% of time and quartile 4 75–100% of time, respectively

Median (IQR) V_t_ was 4.6 (3.3 – 6.2) ml/kg. Nine infants (39%) had a median V_t_ < 4 ml/kg, 12 infants (52%) had a median V_t_ between 4–8 and 2 infants (9%) a median V_t_ > 8 ml/kg. There was a high intra- and interindividual variability in measured V_t_ (Fig. 1). The median (IQR) coefficient of variation was 52 (45 – 77) %. Per infant, a median (IQR) of 36 (25—27) % of inflations were within target range during the duration of PPV, while 42 (25 – 65) % and 10 (3 – 33) % were above and below target range, respectively. Typical RFM waveforms for ventilations within (appropriate ventilations), above (excessive ventilations) and below (inadequate ventilations) the target range are shown in Fig. 2. There were no important changes in V_t_ over the duration of an episode of PPV (Fig. 3; *p* = 0.673).

### Leak and obstruction

Median (IQR) leak was 30 (11 – 44) %. The median number of inflations with obstruction was 0 (0 – 13). At least one obstructive episode was noted in 10/23 (43%) of the infants. Infants with at least one episode of obstruction had a median (IQR) of 9 (1 – 30) obstructed inflations. The infant with the highest V_t_ [13.4 (11.7 – 15.5) ml/kg] had a median leak of 3% and no obstruction (patient 11, Fig. 1). The video recording showed one resuscitator applying the mask tightly with two hands while a second operator applied the inflations to an apneic, floppy infant.

### Clinical outcomes

Ten of 23 (43%) infants included in the study were admitted to the neonatal intensive care unit (NICU) with the following diagnosis on admission: sepsis (*n* = 5), respiratory distress syndrome (*n* = 2), transient tachypnea of the newborn (*n* = 1), hypoglycemia (*n* = 1) and hydrocephalus (*n* = 1). Four of them received non-invasive respiratory support after admission to NICU and two were intubated. V_t_ and number of PPV inflations per infant in those admitted versus those not admitted were similar [V_t_: 4.8 (2.9 – 7.1) ml/kg versus 4.2  (3.4 – 5.9) ml/kg, *p* = 0.95; number of PPV inflations: 58 (31 – 166) versus 58 (32 – 100), *p* = 0.88].

Two infants were intubated (patient 17 and 18). There was a non-significant trend towards a lower V_t_ and a higher number of PPV inflations in those infants who were intubated compared to those who were not [V_t_: 2.6 (1.9 – 3.3) ml/kg versus 5.1 (3.4 – 6.4) ml/kg *p* = 0.07; number of PPV inflations 142 (82 – 201) versus 49 (31 – 103), *p* = 0.24)]. The two infants that were intubated had a higher proportion of inflations with obstruction [19 (7 – 31) %] in comparison with the infants that were not intubated [0 (0 – 1.5) %], albeit the difference did not reach statistical significance (*p* = 0.095).

One of the infants who were intubated had respiratory distress syndrome and the other transient tachypnea of the newborn.

## Discussion

We found marked variability of V_t_ measured in term and late preterm infants when using currently recommended pressure levels for PPV during delivery room stabilization. These findings are in line with recently published data in term infants [7]. Our data, however, highlight that not only was there substantial variability between infants, but V_t_ was inconsistent within the same individual and leak and obstruction were common.

There are several potential causes of the high variability of V_t_ observed in this study: (1) The presence of leak and obstruction may contribute. In this study median facemask leak was 30% and obstruction occurred in 43% of infants. Leak was similar to that previously reported [7]. The incidence of obstruction in term infants has not been reported before. However, obstruction was reported in 25% of preterm infants [5]. (2) The presence of simultaneous spontaneous breaths either during inflation or during deflation may influence V_t_ variability [17]. Spontaneous breaths during PPV can be triggered by tactile stimulation during mask ventilation [18].

Only half of the infants had a median V_t_ within the currently recommended target range of 4–8 ml/kg and V_t_ was in the target rage in only one third of the inflations over the duration of PPV. The optimal V_t_ during mask ventilation at birth is unknown and the currently recommended V_t_ target range of 4–8 mL/kg is largely based on data from endotracheal ventilation [3]. Recent studies suggested that V_t_ in spontaneously breathing term infants during transition are between 2 – 6.5 ml/kg [8] and 2.5 – 8.5 ml/kg [9]. It is unclear whether the suggested reference range of 4–8 ml/kg is safe and effective for term newborns receiving PPV.

The recommended PIP for PPV in the delivery room is based on limited evidence [1] and it remains unclear whether it is sufficient to achieve adequate tidal volumes and lung aeration. Consistent with other reports, our data show that tidal volumes vary considerably despite a fixed PIP [7]. V_t_ tended to be lower in infants who were subsequently intubated. We speculate that higher PIP levels may be necessary in selected critically ill infants, however more data are required. On the other hand, one case showed very high V_t_ under optimal conditions in the absence of leak and obstruction. Based on our data, an individualised approach whereby pressures are adjusted to maintain a safe and effective V_t_ may be beneficial. A RFM in the delivery room may assist in this regard.

Data from term infants who received 20 PPV inflations with a self-inflating bag without a PEEP valve indicate that higher than recommended PIP levels (36 cmH_2_O) were necessary to achieve adequate V_t_ of 3–6 ml/kg [11]. Moreover, there seems to be a positive relationship between heart rate increase and measured V_t_ in depressed infants needing PPV [19].

Clinical assessment of V_t_ and identification of leak and obstructions is challenging, and the use of a RFM may improve the effectiveness of mask ventilation [20]. However, a multicenter randomized controlled trial showed that the use of a RFM compared to no RFM as guidance for V_t_, did not increase the percentage of inflations in a predefined target range [6]. It is unclear whether improvements in monitor design or education in the use of RFM might lead to a different result.

This study has some limitations. It is an exploratory analysis and outcomes were not prospectively defined. It is a single center study with a small sample size and results may not be generalizable to other units using different equipment. Different definitions for target range for V_t_ as well as leak and obstruction used in the literature make comparisons with other studies difficult. It would have been interesting to evaluate the effectiveness of the resuscitation in terms of heart rate and oxygen saturations between the groups according to tidal volume, but we were unable to measure these data consistently in our study population.

## Conclusion

Tidal volumes delivered to term and late preterm infants in the delivery room vary widely both between and within infants, despite a consistent PIP. Airway obstruction and facemask leak are common, resulting in only one third of inflations having an expired V_t_ in the recommended range. Finding optimal pressure settings seems challenging and a respiratory function monitor may improve the safety and effectiveness of mask ventilation.

## Data Availability

De-identified individual participant data, study protocol and statistical analysis data are available from three months to 10 years following article publication to researchers who provide a methodologically sound proposal, with approval by an independent review committee (“learned intermediary”). Proposals should be directed to janine.thomann@usz.ch to gain access. Data requestors will need to sign a data access or material transfer agreement approved by USZ.

## Abbreviations

V_t_: Tidal volume
PPV: Positive pressure ventilation
IQR: Inter quartile range
RFM: Respiratory function monitor
PIP: Peak inspiratory pressure
